# Compliance and determinants of infection prevention and control practices among sanitary workers in public hospitals, Eastern Ethiopia: A cross‐sectional study

**DOI:** 10.1002/hsr2.2318

**Published:** 2024-08-23

**Authors:** Sina T. Tolera, Nega Assefa, Abraham Geremew, Elka Toseva, Tesfaye Gobena

**Affiliations:** ^1^ Haramaya University College of Health and Medical Sciences Harar City Ethiopia; ^2^ Department of Hygiene, Faculty of Public Health Medical University of Plovdiv Plovdiv City Bulgaria

**Keywords:** compliance, control, determinants, hospital, infection prevention, sanitary workers

## Abstract

**Background:**

Best practice of infection prevention and control (IPC) is a hallmark for the patient care in health care settings, but it is a major problem in developing countries like Ethiopia where resources are limited. Ethiopia Federal Ministry of Health working to strengthen its IPC program, but still it there is no organized study conducted on assessment of performance gaps and implementation challenges of IPC practice faced by hospital staffs particularly among sanitary workers (SWs) at public hospitals inline to national and international guidelines.

**Aim:**

This study focuses on compliance and determinants of IPC among sanitary worker in public hospitals in eastern Ethiopia: A cross sectional study design.

**Method:**

A cross‐sectional study was conducted among 809 SWs and eight IPC experts in public hospitals, eastern Ethiopia, from May to August 2023. A standard questionnaire was used to collect data. Face‐to‐face interview was conducted. Ten (10) question pursued to answer YES/NO were prepared. The cut point for categories of IPC practice was 1: *Good* (16–20 scores), 2: *Fair* (10–15 scores), and 3: *Poor* (<10 scores). The cut point for compliance and noncompliance of IPC practice among hospitals was mean (500.1). Multi‐level ordinal logistic regression models was applied to explore the association of dependent and independent variables at individual level (Model 1), hospital level (Model 2) and at both (Model 3). Crude odds ratio (COR) and adjusted odds ratio (AOR) at 95% confidence interval (CI) were used to report the result.

**Result:**

The compliance of IPC practice among SWs was 36.21% (32.72, 39.82%). The Multilevel ordinal logistic regression model shows that SWs who have good knowledge of IPC trend (AOR: 4.70, 95% CI: 2.11–10.46), SWs who are not addictive with alcohol (AOR: 2.35, 95% CI: 1.15,4.78) and chew Khat (AOR: 1.62, 95% CI: 1.06,2.46) and smoke cigarette (AOR: 3.15, 95% CI: 2.35–5.41), and SWs without job stress (AOR: 1.46, 95% CI: 0.86–2.48) were more compliant to IPC practice. Similarly, those who do not have workload (AOR: 2.74, 95% CI: 1.56–4.82), work <8 h/day (AOR: 1.46, 95% CI: 0.92–2.30), and those who have good social recognition in hospitals (AOR: 6.08, 95% CI: 4.24–8.71) were more likely to increase the compliance of IPC practice among SWs. The multilevel random‐effect model revealed 93.71% of the variability of compliance of IPC practice explained by both individual and hospital level factors.

**Conclusion:**

The overall study found that inadequate IPC practice was reported among SWs as well as by IPC experts due to poor knowledge of IPC trend and individual behaviors and working environment. Thus, the study advised that hospitals have to develop and establish IPC implementation guidelines in order to solve the concerns among these groups; national IPC office should follow its implementation across health care settings particularly at public hospitals.

## 1 INTRODUCTION

Best practice of infection prevention and control (IPC) is a hallmark for the patient care in health care settings. IPC is designed for prevention of infections that may acquire within health care institutions.^1^, ^2^ Consequently, IPC is a set of scientifically validated procedures designed to stop infections from harming patients, healthcare providers, and the general public.^1^ The reality is that IPC has only recently fully recognized the importance of the hospital environment for staff protection and patient care. There is currently adequate evidence demonstrating that designed IPC and maintaining a clean hospital environment in the health care settings are significant contribution for reducing infection rates.^2^

The study found that poor IPC practice due to healthcare‐acquired infections (HAIs) has a number of negative effects on health and raises the possibility of long‐term incapacity within health care settings.^3^ The poor IPC are due to longer hospital admissions, antibiotic resistance,^4^ lack of leadership commitment, financial strain in the health care systems,^5^ and exorbitant expenses for patients and their families, and preventable deaths.^5^ Every year, one in 10 of the world's population visits health care institutions and is infected with HAIs due to improper waste management, improper personal protective equipment (PPE) practice and lack of well procedure.^6^ As the result, HAIs are cross‐cutting challenges in health care institutions that no organization or country can claim to have solved and impacted on the hundreds of millions across the globe.^7^ Over 90% of these HAIs occur in developing or low‐income countries, primarily in Africa, where there may be a lack of attention to advised precautions.^3^

A lot of study found that healthcare staffs including sanitary workers (SWs) in numerous developing countries do not follow IPC protocols due to underestimation for the risk of infections, lack of or low awareness of the staffs, low education, and lack of budget to prevention and control of HAIs.^4^ These areas struggled with unsafe needle injections, scare of budget, inadequate medical waste management, and lack of PPE supply, which are core components of IPC.^8^

As a result, sanitation workers in developing or low‐income countries sometimes neglect their responsibility to uphold a safe and sanitary workplace. SWs are the individuals working on poor condition of service and poor adherence to occupational health and safety practices in institutions, in which worse cases or scenarios may be bound to happen elsewhere.^9^ On the other hand, these workers may be exposed or get contaminated indirectly through contact with contaminated ambient surfaces or directly through direct contact with patients. This is the main method that illnesses acquired via medical care are transmitted. Between 50% and 70% of HAIs are thought to be the result of contaminated hands and 30%–50% are associated with transmission through the environment.^2^

Nationally, Ethiopia's Federal Ministry of Health is stepping up its national efforts to implement good IPC and provide a safe environment to create comfortable work environment free from hazards, as well as a health care setting that is comfortable and safe for patients, staff, guests, attendants, and communities—all of whom are crucial factors in determining the quality of healthcare.^10^ However, inadequate funding for IPC service, as well as environmental sanitation and hygiene service, poses long‐term risks to healthcare personnel.^2^ The most common infections in the health care settings due to poor IPC practice in the health care settings are HAIs.^11^, ^12^

A lot of tasks in IPC activities are performed in healthcare facilities by sanitation workers or staffs. In this case, SWs are typically made up of cleaners, waste collectors, and waste disposal workers. These are nonmedical staff members who are in charge of arranging and cleaning hospital wards, disposing of garbage, and organizing and washing linens. Because of these work characteristics, there is a risk of infection.^13^ Health care personnel—especially SWs like cleaners, medical waste collectors, and pesticide sprayers—must adhere to IPC protocols.^2^, ^14^, ^15^ On the other hand, this is due to evidence that IPC practices, especially those involving cleaning and environmental hygiene, can lower hospital acquired infections (HAIs) rates.^14^ This is due to the fact that protecting hygienic staff at health care facilities also entails protecting patients, healthcare professionals, guests, students, and the local community from potentially fatal infectious infections.^15^

However, SWs always perform in locations where they are in close touch with both patients’ bed and medical waste in their routine works. That is why these groups are facing with multiple burden of infection and other occupational related problems.^16^, ^17^ Due to a lack of resources, inadequate sanitation, and poor hygiene practices, IPC measures have been ignored, which has resulted in a high burden of HAIs, as evidenced by available data.^18^ According to the study, IPC is a catch‐all phrase for policies, procedures, protocols, and tactics that may reduce the risk of infection and the spread of microorganisms in healthcare settings. IPC are necessary to provide the facility sector with safe and excellent services.^4^ As evidence based on research, best practices for IPC promote medical providers delivering safe, high‐quality care to patients, visitors, employees, and the environment of healthcare.^19^

In hospital settings, there is a considerable risk of infection via blood, body fluids, or contaminated objects when IPC procedures are not followed correctly. In accordance with the IPC, one must observe hand hygiene, respiratory and cough hygiene, PPE, safe medical equipment, safe environmental management, laundry management, spill control for blood and bodily fluids, and waste management in public hospital.^20^ Numerous study have shown that inadequate attention to IPC training results in poor IPC practices.^21^ In addition, there was no formal reporting system of IPC in health care settings,^22^ did not have postexposure prophylaxis or follow proper IPC practices,^6^ did not follow up on following IPC practices,^4^ did not have any education or campaigns,^23^ did not integrate IPC activities,^24^ and did not show commitment from managers.^25^ A lot of studies also showed that certain variables worsening these difficulties are attributable to poor IPC practice compliance.^26^, ^27^, ^28^

However, compliance with IPC practices among SWs are still not well integrated into healthcare facilities as a whole, especially in public hospitals. In addition, there was no prior research to determine the extent of IPC practice in accordance with World Health Organization (WHO) guidelines,^29^ nor did they identify the level of IPC practice among SWs in public hospitals. Moreover, Ethiopia Federal Ministry of Health is working to strengthen its IPC program, but until recently has lacked an assessment of performance gaps and implementation challenges faced by hospital staffs particularly among sanitary workers. Thus, the purpose of the current study was to assess compliance of IPC practices as well as factors influencing its practice among SWs in public hospitals in eastern Ethiopia.

## 2 METHODS

### 2.1 Settings and study period

Hospital‐based cross‐section study was conducted in eight selected public hospitals in eastern Ethiopia from May 1, 2023 to August 30, 2023. Eastern Ethiopia was purposely selected from the part of Ethiopia. At Stage 1, Eastern Ethiopia was divided into four states: Oromia state, Harari Regional State, Somali Regional state, and Dire Dawa city administrative. Then eight public hospitals were selected at random from a total of 14 public hospitals, with an equal probability allocated to each region. Hiwot Fana Comprehensive Specialized Hospital (HFCSH), Jugola General Hospital (JGH), Dilchora Referral Hospital, Sabian General Hospital (SGH), Jigjiga University Sheik Hassan Referral Hospital (JUSHRH), Karamara General Hospital (KGH), Bisidimo General Hospital (BGH), and Chiro General Hospital (CGH).

**Figure 1:**
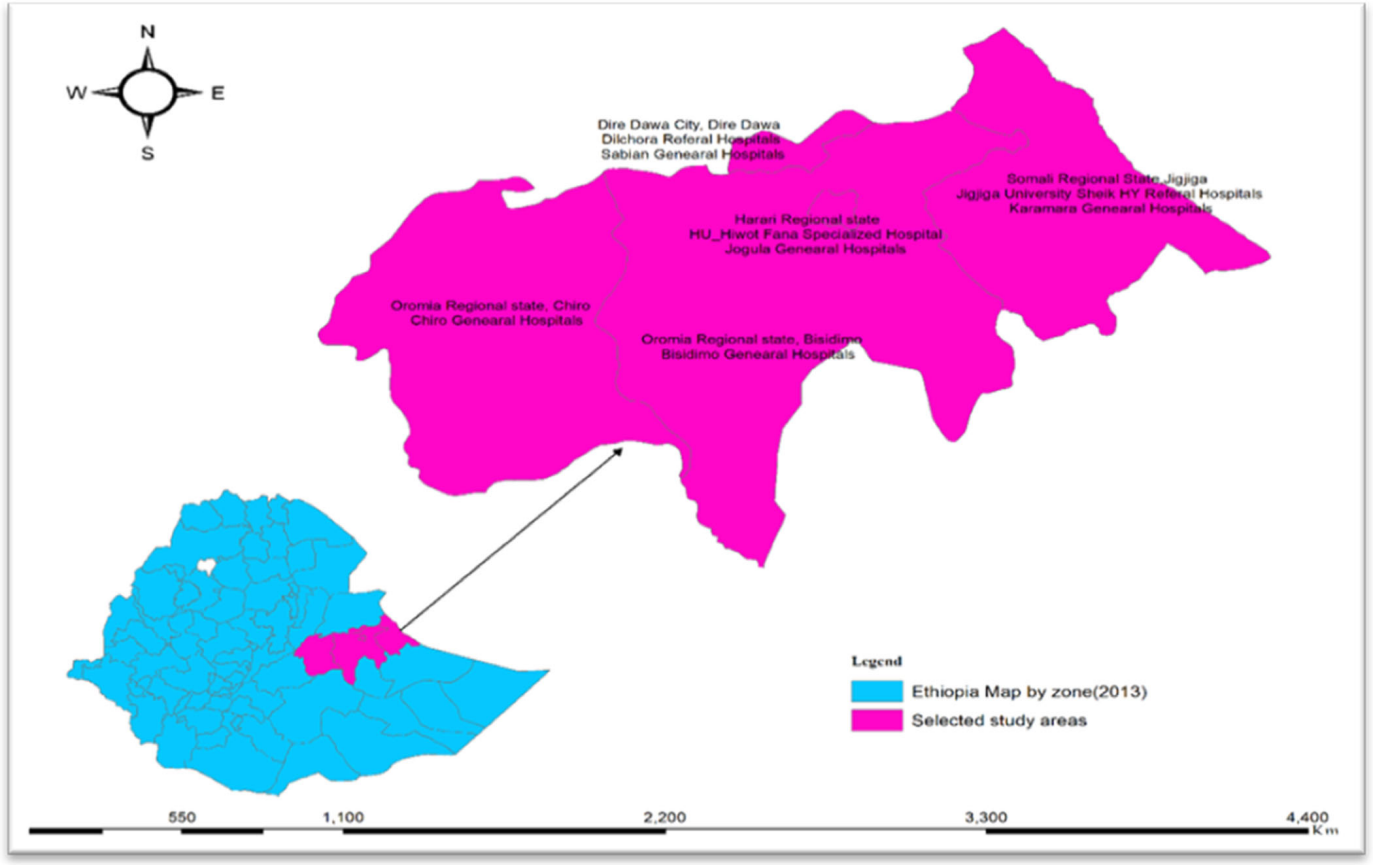
Map of study areas for compliance and determinants of infection prevention and control among sanitary workers created using ARCGIS software from Ethiopia geographic information system.^32^

The mean and SD (mean ± SD) by bed occupancy in eight hospitals was 269.5 ± 132.6) per a day. Meanwhile, mean ± SD for outpatient flow and inpatients in these selected hospitals were 154.4 ± 67.4 and 233.9 ± 125.4, respectively. As the result, a mean ± SD total of eight outpatient flow and inpatients are 388.3 ± 190.^30^ As human power capital for the eligible hospitals, about 5680 hospital staff are working in the different department of these hospitals.^31^ Moreover, in these hospitals, about 809 sanitary workers, namely cleaners and waste collectors, are found. Therefore, eight public hospitals have been selected from Eastern Ethiopia as shown in Figure 1.

### 2.2 Study design

A cross‐sectional research design was conducted among SWs in selected public hospitals in Eastern Ethiopia from May to August, 2023.

### 2.3 Study variables

#### 2.3.1 Dependent variables

Compliance infection prevention, and control practice. It is defined as a bundle of 10 practical protocol and produces that SWs should follow during their engagement with hygiene and sanitation of hospitals, to prevent exposure to infection, antimicrobial resistance, and occupational infections from themselves, patients, healthcare workers, and the community within the hospitals.^33^ Ten principal items were derived from the World Health Organization (WHO) guideline for IPC practice^29^ and were rated by Boolean logic (YES [1]/NO [0]) by each participant. In addition to compliance by hospitals that consisted eight core components, 93 scores were evaluated by IPC experts, which is, adopted from WHO guideline for IPC practice.^29^

#### 2.3.2 Independent variables

Independent variables included demographic characteristics such as gender, age, educational status, working experience, type of job categories, marital status, and salary. Institutional and safety variables included supply and availability of PPE, occupational health and safety (OHS) training, and level of IPC practice. Behavioral variables included consumption of alcohol, smoking cigarette, khat chewing (local name, it is a green leaf used as addictive) work/job stress, and sleeping disorder. Environment/work pattern variables included satisfaction of environmental and job, daily working hours (either >8 h/day or <8 h/day), workload, and social recognition (either bad or good).

### 2.4 Sample determination

The sample size for compliance of IPC among sanitary workers was estimated using single proportion formula, the reliability coefficient at 95% confidence interval (95% CI: 1.96), *p* is the population proportion, *q* is equal to 1 − *p*, and *d* is the acceptable error (0.05). The prevalence of compliance of IPC practice was 47.7%.^15^ Thus, the sample size (ni) become 383. Using design effect of 2.00, the sample size was 766. Adding with 5% contingency (38 individual), the final sample size was 804. This figure is approaching to all sanitary workers in eight hospitals (*N* = 809). Thus, all units were recruited for the final study.

### 2.5 Selection procedures

In this study, eight public hospitals were selected at random from a total of 14 public hospitals located throughout the regional states, with an equal probability allocated to each study location. Accordingly, a total of 234, 175, 82, and 318 sanitary workers were eligible from HFCSH and JGJ (Harari regional state), BGH and CGH (Oromia regional state), DRF and SGH (Dire Dawa city administration), and from JUSHRH and KGH (Somali regional state), respectively.

### 2.6 Assessment tools

#### 2.6.1 IPC items for workers

Ten closed‐ended questions derived from the eight core IPC program questions, which was adapted from WHO guideline for IPC.^29^ All prepared items were prepared by modifying Johns Hopkins University School of Hygiene and Public Health Safety Climate Questionnaire.^34^ The items were measured by Boolean logic (YES [1]/NO [0]), adapted from Speziale and Carpenter.^35^ As the result of dichotomous answers, the overall answer evaluated from 20 scores. Accordingly, the level of IPC practice classified as good IPC practice for SWs accounted 16–20 score out of 20 score, Fair IPC practice for those accounted 10–15 score; and Poor IPC practice (noncompliance) those accounted <10 scores, adapted from the previous studies.^15^, ^36^

#### 2.6.2 Associated factors items

Eleven questions were prepared and assessed them using Boolean Logic (YES [1]/NO [0] prepared. For those who answered “Yes,” they have been selected as: 1: *seldom/monthly*; 2: *occasionally/weekly*; 3: *frequently/daily*.^37^

#### 2.6.3 IPC Practice items for expert evaluation

In eight hospitals, eight IPC experts were recruited for data collection for IPC evaluation. To determine the level of IPC practice among hospital, the tool adoption from “Infection Prevention and Control Framework” scoring system was used. IPC^29^ having a closed‐ended, well‐organized survey was administered. Experts evaluated the eight IPC core programs from 800. Of this, Core 1 (i.e., existence of IPC program) contains 120 score, Core 2 (IPC Guideline) = 102.5 score, Core 3 (IPC training) =146 score, and Core 4 (HAIs) = 110 score. In the same manner, Core 5 (Multimodal strategies) contains 125 score, Core 6 (Monitoring and feedback) = 102.5 score, Core 7 (Workload and staffing) = 140 score), and Core 8 (Build IPC environment) = 138.5 score, adapted from WHO.^29^ Then from overall 800 points (because there were eight programs and each has 100 score), the ranges were set. Accordingly, the ranges of 0–200 if inadequate IPC practice, 201–400 if Basic IPC practice, 401–600 if intermediate IPC practice, and 601–800 if advanced IPC practice.^29^ From these group data, the mean IPC practice was 500.1. Therefore, it was used for cut point of compliance if adhered, or good or adequate IPC service, while it is noncompliance IPC practice if it is nonadhered, or poor or inadequate.

### 2.7 Data collection methods

Eight data collectors participated in data collection. All of them have Master degree in public health, occupational health, and environmental health. Data collection was carried out face‐to‐face by data collectors. The pattern of the study was carried out into three types of shifts or job rotation for consecutive 3 months: the first shift (Morning), second shift (Afternoon), and third shift (night). All hospital sanitary workers worked within the rotation of three shifts per week. The first shift starts at 7:00 a.m. and ends at 12:00 a.m. The second shift starts at 1:00 p.m. and ends at 5:00 p.m. The third shift starts at 12:00 p.m. and ends at 6:00 a.m. This group works on schedule for two consecutive days and then rests for 2 days. By considering this, the questionnaires were administered (Morning: 9:00–10:00 a.m.) for Shift 1. For Shift 2, the interview was done (Afternoon: 3:00–4:00 p.m.). The same procedure was done for Shift 3 after 2 days.

### 2.8 Data analysis

The data was entered into Epi Data version 3.1 (Model type 27jan2008). Then it was exported to Stata 17 MP version (StataCorp). Descriptive statistic was used to characterize one independent or dependent variable. Also, it was performed by Microsoft Excel office 19 (Microsoft Corporation) and Med‐Calc. version 22.021. The information was displayed using the mean, SD, percentage (%), and mean ± SD, to illustrate the trends in IPC core compliance at each facility. Meanwhile, to explore relationship of compliance with IPC practice and independent variables, multilevel ordinal logistic regression model was used. Four models were performed: Model 0 (Null model), Model 1 (for individuals’ variables), Model 2 (for hospital variables), and a combination of Model 1 and 2 (Model 3). Model 0, Model 1, and Model 2 were not presented, to limit the number of tables, and thus only Model 3 table was presented. The crude odds ratio (COR) and adjusted odds ratio (AOR) with a 95% CI were presented with two‐sided level of significant. The COR and AOR with a 95% CI were presented at Model 3. Independent variables having a *p* value of 0.20 were candidate for the final analysis and variables with AOR with (95% CI) at a *p* < 0.05 were reported. Intraclass correlation coefficient (ICC), Akaike information criterion (AIC), Bayesian information criterion (BIC), likelihood ratio, sensitivity, and specificity were computed as well, to test model for multilevel analysis. Then, the optimal model that has the largest likelihood ratio at each model was selected. The multicollinearity was also examined using the variance inflation factor (VIF), which measured how an independent variable's variance is inflated in each other, with a cutoff point of <10. Also, Hosmer and Lemeshow (HL) goodness‐of‐fit test was used for model fit, where HL with a small *χ* ^2^ values with larger *p* value closer to 1 or >0.05 was accepted according to validated study.^38^

### 2.9 Data quality

A survey of the literature on objective acquiescence served as a reference for developing the questions. First, conventional and structured questionnaires that are applicable in high‐income, middle‐income, and low‐income nations were employed to guarantee the quality of the data. Four supervisors, eight data collectors, and the second data assurance were hired from relevant professions. Third, data collectors received the necessary training (including instruction on how to deal with the data gathering process and research areas, including top management in the event of an unsatisfactory review). Before the main research, pretest was conducted on 5% of SWs at Haramaya General Hospital.

### 2.10 Ethical statement

The study was approved by the Haramaya University College of Health and Medical Sciences, Institutional Health Research Ethics Review Committee (Reference from Ethics: IHRERC/064/2023). Then, Haramaya University research affair wrote an official letter to eight selected hospitals to carry out this study. Then, all hospitals administrator showed their willingness and gave permission of the institution. Then, the selected hospitals signed institutional consent with principal investigator for the study or research to be carried out in their hospitals. For the participant consent, the participants were competent adults, ages >18 years. As a result, all participant provided consent. The questionnaire was coded to prevent names from appearing. All the information supplied was kept private and secure according to World Medical Association's Helsinki. It means there is no information that can be used to specifically identify the participants. The study's findings apply to the entire study group and are not particular to any one individual.

## 3 RESULT

### 3.1 Demographic variables

Out of 809 of sanitary workers, 729 (90.11%) of them responded. The majority were females (98.49%), between the ages of 24 and 35 years (48.01%), married (69.41%), cleaners (93.14%), permanents (97.26%), and shift one (49.38%). The mean ± SD for age (years) and work experience (years) were 34.35 ± 7.60 and 6.65 ± 6.36, respectively. Similarly, educational status (grade) and monthly income were 6.78 ± 2.51 and $36.32 ± 6.68 (1982 ± 365ETB), respectively (Table 1).

**Table 1:** Socio‐demographic status of SWs in selected public hospitals in Eastern Ethiopia, 2023.

Socio‐demographic	Classification	Frequency (No)	Percentage (%)	Mean ± SD
Sex	Female	718	98.49	
	Male	11	1.51	
Age	≤24 years	63	8.64	
	25–35 years	350	48.01	34.35 ± 7.60
	>35 years	316	43.35	
Work experience	≤2 years	133	18.24	
	3–5 years	288	39.51	6.65 ± 6.36
	>5 years	308	42.25	
Educational status	≤Grade 4	160	22.07	
	Grade 5–8	283	39.03	6.78 ± 2.51
	>Grade 8	282	38.90	
Marital status	Single	142	19.48	
	Married	506	69.41	
	Separated	59	8.09	
	Divorced	22	3.02	
Income monthly salary (USD [$])	≤$20.15^a^	12	1.65	
	$20.16–42.95^b^	672	92.18	36.32 ± 6.68
	>$42.95	45	6.17	
Job categories	Cleaners	679	93.14	
	Waste collectors	50	6.86	
Employment type	Permanents	709	97.26	
	Contracts and other	20	2.74	
Type of shift during data collection	Shift 1	360	49.38	
	Shift 2	262	35.94	
	Shift 3	106	14.54	

*Note*: Levels of civil service salary by National Job Evaluation and Grading, 2019, where $1 = 54.58 ETB, July, 2023.

^a^ Level salary I ($20.15 = 1100 ETB).

^b^ Level V ($42.95 = 2344 ETB).

### 3.2 Compliance and determinants of IPC practice

The current study found that the level of IPC practice among SWs selected public hospitals were 264 (36.21%), 197 (27.02%), and 268 (36.76%) for good, fair, and poor, respectively. A total of 17 determinants that were statistically significant (*p* < 0.20) were selected for the final candidate for AOR of multilevel ordinal logistic. Accordingly, the model shows that SWs who did not get OHS/IPC training (AOR: 0.30, 95% CI: 0.20,0.43), no availability and supply of PPE (AOR: 0.80, 95% CI: 0.57,1.130) were more likely to lower the compliance of IPC practice. However, those who have good knowledge of IPC practice (AOR: 4.70, 95% CI: 2.11–10.46), who were not addictive with alcohol (AOR: 2.35, 95% CI: 1.15–4.78), and chew Khat (AOR: 1.62, 95% CI: 1.06,2.46) and cigarette smoking (AOR: 3.15, 95% CI: 2.35–5.41), and those who did not have job stress (AOR: 1.46, 95% CI: 0.86–2.48) were more likely to increase the compliance of IPC practice as compared to their counterparts (Table 2). Similarly, SWs who worked <8 h/day (AOR: 1.46, 95% CI: 0.92–2.30), no workload (AOR: 2.74, 95% CI: 1.56–4.82), and social recognition (AOR: 6.08, 95% CI: 4.24–8.71) were more likely to report high IPC compliance (Table 2).

**Table 2:** Model 3 multilevel ordinal logistic regression model for predictors of IPC practice among SWs in public hospitals, Eastern Ethiopia, 2023.

		Level of IPC practice (*N* = 729)				
Categories of variables	Categories of variables	Poor: 268	Fair: 197	Good: 264	COR (95% CI)	AOR (95% CI)
Individual variables						
Knowledge	Poor	215 (80.22)	90 (45.69)	236 (89.39)	1 (Reference)	1
	Fair	50 (18.66)	95 (48.22)	4 (1.52)	0.54 (0.39,0.74)*	0.85 (0.55,1.30)
	Good	3 (1.12)	12 (6.09)	24 (9.09)	2.94 (1.56,5.57)*	4.70 (2.11,10.46)**
Education	<Grade 4	52 (19.70)	3 (1.52)	105 (39.77)	1	1
	Grade 5–8	114 (43.18)	60 (30.46)	109 (41.29)	0.38 (0.26,0.57)*	0.60 (0.38,0.95)***
	>Grade 8	98 (37.12)	134 (68.02)	50 (18.94)	0.29 (0.19,0.43)*	0.45 (0.28,0.72)**
Sleeping disorder	Yes	43 (16.04)	72 (36.55)	82 (31.06)	1	1
	No	225 (83.96)	125 (63.45)	182 (68.90)	0.56 (0.42,0.75)*	0.42 (0.29,0.61)****
Heavy alcohol consumption	Yes	40 (14.93)	10 (5.08)	7 (2.65)	1	1
	No	228 (85.07)	187 (94.92)	257 (97.35)	4.56 (2.55,8.13)*	2.35 (1.15,4.78)**
Chew khat	Yes	74 (16.04)	10 (37.56)	127 (3.79)	1	1
	No	225 (83.96)	123 (62.44)	254 (96.21)	1.76 (1.27,2.44)*	1.62 (1.06,2.46)**
Smoking tobacco	Yes	32 (11.94)	25 (12.69)	9 (3.41)	1	1
	No	236 (88.06)	172 (87.31)	255 (96.59)	2.15 (1.35,3.41)*	3.15 (2.35,5.41)****
Job stress	Yes	120 (44.78)	78 (39.59)	13 (4.92)	1	1
	No	148 (55.22)	119 (60.41)	251 (95.08)	4.67 (3.42,6.37)*	1.46 (0.86,2.48)**
Employment	Permanent	254 (94.78)	191 (96.95)	264 (100)	1	1
	Contract	14 (5.22)	6 (3.05)	0 (0.00)	0.20 (0.08,0.52)*	0.41 (0.14,1.22)****
Hospital variables						
OHS/IPC training	Yes	137 (51.12)	83 (42.13)	212 (80.30)	1	1
	No	131 (48.88)	114 (57.87)	52 (19.70	0.39 (0.29,0.51)*	0.30 (0.20,0.43)**
Have workload	Yes	110 (41.04)	48 (24.37)	9 (3.41)	1	1
	No	158 (58.96)	149 (75.63)	255 (96.59)	5.93 (4.16,8.45)*	2.74 (1.56,4.82)****
Working more than 8 h/day	Yes	95 (35.45)	76 (38.58)	9 (3.41)	1	1
	No	173 (64.55)	121 (61.42)	255 (96.59)	3.69 (2.69,5.07)*	1.46 (0.92,2.30)***
Safe environmental	Yes	259 (96.64)	178 (90.36)	231 (87.50)	1	1
	No	9 (3.36)	19 (9.64)	33 (12.50)	2.53 (1.54,4.15)*	1.55 (0.70,3.46)
Bad hospital recognition	Yes	221 (82.46)	82 (41.62)	111 (42.05)	1	1
	No	47 (17.54)	115 (58.38)	153 (57.95)	3.83 (2.88,5.08)*	6.08 (4.24,8.71)**
Unavailability lack of PPE supply	Yes	128 (47.76)	71 (36.04)	189 (71.59)	1	1
	No	140 (52.24)	126 (63.96)	75 (28.41)	0.47 (0.36,0.62)*	0.80 (0.57,1.13)***
Job rotation	Shift 1	132 (49.25)	106 (53.81)	122 (46/21)	1	1
	Shift 2	108 (40.30)	63 (31.98)	91 (34.47)	0.92 (0.68,1.23)	0.60 (0.43,0.85)****
	Shift 3	28 (10.45)	28 (14.21)	51 (19.32)	1.70 (1.14,2.54)*	0.99 (0.62,1.60)
Model summary	ICC	AIC	BIC	LR	Sensitivity	Specificity
Model 1	91.64%	492.33	556.54	0.11	92.84%	41.67%
Model 2	92.64%	468.90	519.41	0.15	87.20%	57.46%
Model 3	93.71%	447.22	552.70	0.23	86.98%	68.94%

*Note*: At Model 3: HL goodness *χ* ^2^ = *p* = 0.055, indicating it acceptable; b/s > 0.05; VIF: 3.62, indicating it is acceptable if b/s is <10; ICC, 39.55% of the variance was between SWs among hospitals; Likelihood ratio: 0.093, indicating the deviance of Model 3 from the Model 0 was 0.093; Sensitivity = 80.72%, indicating that the model's ability predicted 80.72 of true positive. An estimate of a constant plus the relative distance between the unknown true likelihood function of the data and the fitted likelihood function for AIC was 447.22, whereas an estimate of a function of the posterior probability of being true, under a certain Bayesian setup for BIC was 552.70. As detailed above, all evidence of statistical output showed that there was good‐of‐fit of model.

Abbreviations: AIC, Akaike information criterion; BIC, Bayesian information criterion; ICC, intraclass correlation coefficient; LR, likelihood ratio; OHS, occupational health and safety.

* Statistical significance (SS) of *p* < 0.20.

** *p* < 0.001

*** *p* < 0.01

**** *p* < 0.05.

### 3.3 Compliance of IPC practice by main cores

**Figure 2:**
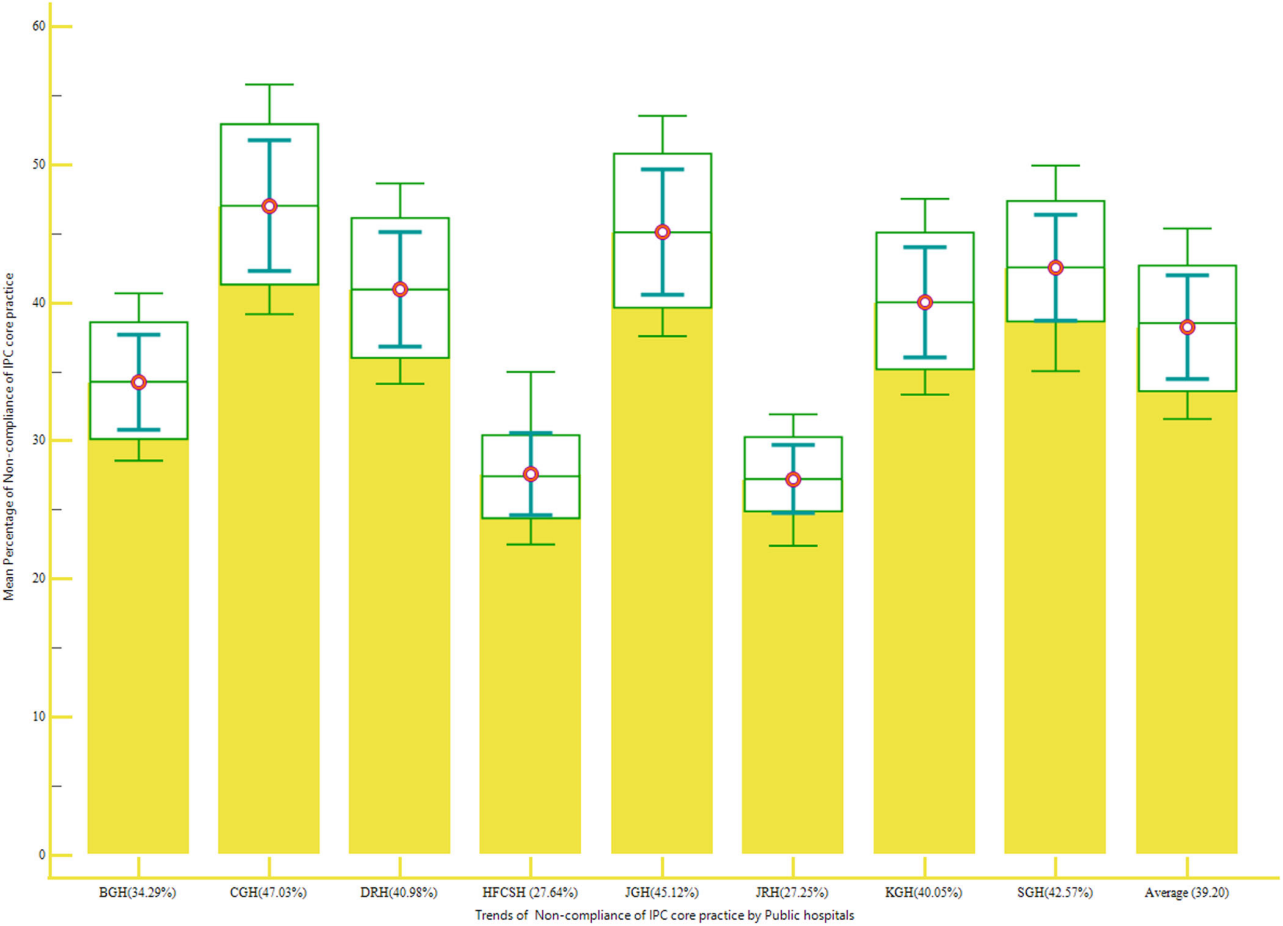
Noncompliance of infection prevention and control (IPC) practice by core among hospitals, 2023. BGH, Bisidimo General Hospital; CGH, Chiro General Hospital; DRH, Dilchora Referral Hospital; HFCSH, Hiwot Fana Comprehensive Specialized Hospital; KGH, Karamara General Hospital; SGH, Sabian General Hospital.

The recent survey indicated that Compliance of IPC practice by main cores among selected hospitals was 60.82%. That means the nonadherence with IPC measures in selected public hospitals was 39.18%, based on a report by IPC professionals. Of this, 45.4%, 44.32%, and 42.6% were accounted for noncompliance with IPC due to training, noncompliance due to building environmental for IPC and noncompliance with IPC due to workload and staffing cores were shared, respectively (Figure 2).

### 3.4 Compliance of IPC practice by hospitals

**Figure 3:**
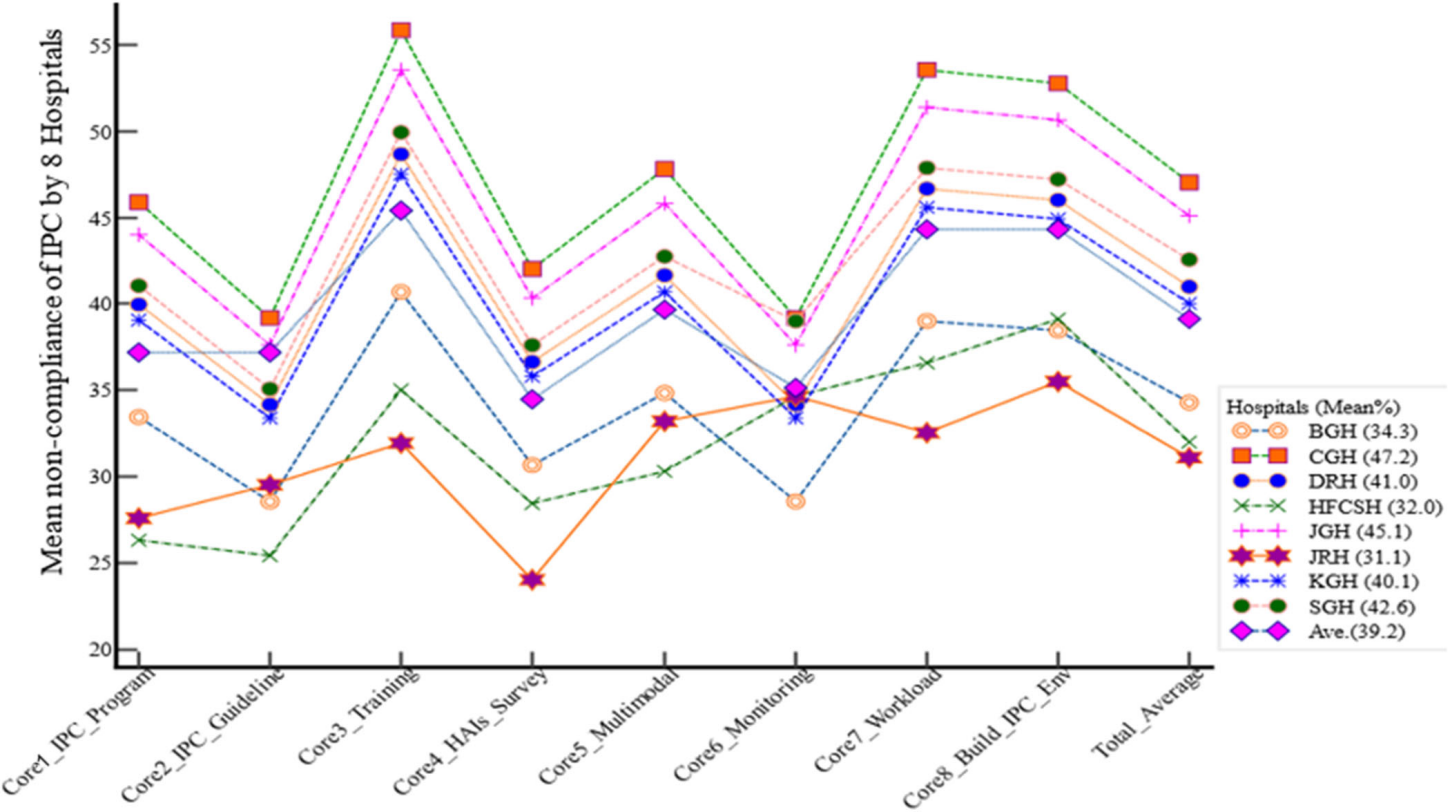
Noncompliance of infection prevention and control (IPC) trends by selected hospitals, 2023. BGH, Bisidimo General Hospital; CGH, Chiro General Hospital; DRH, Dilchora Referral Hospital; HFCSH, Hiwot Fana Comprehensive Specialized Hospital; KGH, Karamara General Hospital; SGH, Sabian General Hospital.

From eight selected public hospitals, the three leading prevalence of noncompliance of IPC among selected public hospitals were accounted as 47.2%%, 45.1%, and 42.6% for CGH, JGH, and SGH, respectively. On the other hand, the compliance of IPC practice for CGH, JGH, and SGH was 52.8%, 54.9%, and 57.4%, respectively (Figure 3).

### 3.5 Compliance of IPC by building IPC environment

**Figure 4:**
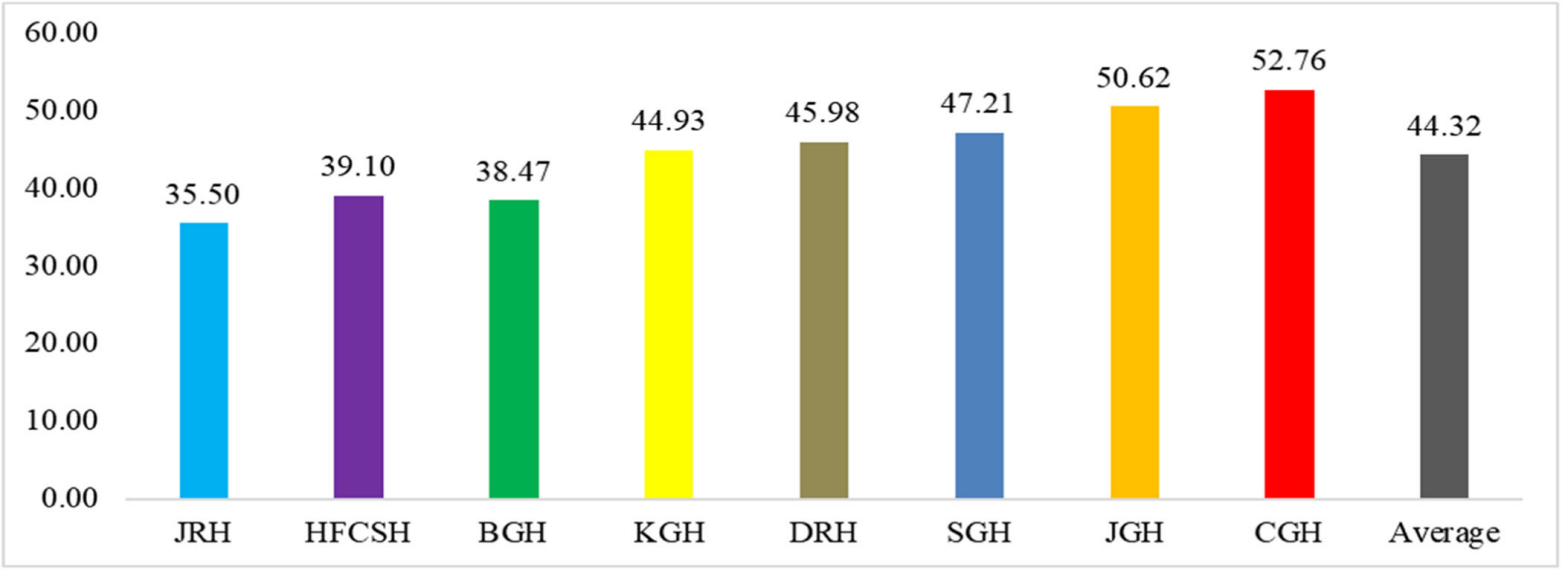
Noncompliance of infection prevention and control (IPC) practice by building environmental situation among public hospitals, 2023. BGH, Bisidimo General Hospital; CGH, Chiro General Hospital; DRH, Dilchora Referral Hospital; HFCSH, Hiwot Fana Comprehensive Specialized Hospital; KGH, Karamara General Hospital; SGH, Sabian General Hospital.

The average prevalence of noncompliance and compliance of IPC by building environmental situation for IPC activities among selected public hospitals in eastern Ethiopia was 44.32% and compliance was 55.68% (Figure 4).

## 4 DISCUSSION

The current study survey showed that the noncompliance of IPC practice among SWs in public hospitals found in eastern Ethiopia was 36.76%. In contrast, it is higher than 23.00% reported by Nelson et al.,^7^ 12.5% reported by Oppong et al.,^39^ and 15.0% reported by Yohannes et al.^40^ The dissimilarity may be attributable to the intended studied units (i.e., in the current study, hospital SWs were the target study group [Table 2], whereas in Yohannes et al.^40^ were health care workers (HCWs). The second possible reason was this might be due to IPC training or the understanding of IPC practice between SWs and HCWs. Additionally, it might be due the fact that acute hospitals’ staffs such as general, referral, and teaching hospitals, have a higher degree of IPC knowledge as compared to other healthcare facilities. However, it was <46.2% reported by Daba et al.,^41^ 47.70% reported by Tesfaye et al.,^15^ and 64.30% discovered by Belay et al.^42^ The discrepancy may have been caused due to the measurement instrument that was utilized by the researchers. That means, the current study used <50% cut point for compliance of IPC practice questions. However, the cut point for the prepared questions was 50% for the study,^41^ 70% for Rutala et al.,^43^ and 75% for Belay et al.^42^

To investigate the associated factors that might be hindered, the adherence of IPC practice between the hospitals as well as between the individuals and multilevel ordinal logistic regression model was applied. Accordingly, the model demonstrated that SWs who have good knowledge of IPC were more likely to increase IPC practice compliance by 4.70 times when compared to those with weak PPE utilization knowledge. The current study similar to the previous study, where good awareness towards IPC were more likely to increase the level of IPC practice by 1.54 times as compared to poor knowledge.^15^ The other study also found that workers who were well‐informed about IPC were more likely to enhance IPC compliance by a factor of 3.13 times as compared to less informed towards IPC practice.^40^

The model also found that SWs without job stress were 1.46 times more likely to increase IPC practice compliance than those with job stress. The study found that job stress has significant effect on physical and mental health of the workers, to properly follow the work procedures.^44^

In a similar manner, SWs who have no workload were more likely to be compliant with IPC practices by 2.74 times than SWs with heavy workloads. The other study found that those haven't workload 2.25 time as compared those have it.^45^ This implies that reduced workload and more efficient work organization should contribute to the improvement of IPC protocols.^45^ Also, SWs who worked <8 h per day were more likely to report IPC practices as complaints by 1.46 times than SWs who worked >8 h per day. This implies that workers with daily work durations exceeding 8 h were more likely to exhibit poor compliance with IPC compared with those working 8 h or less per day.^41^ The analysis also found that compared to those without social recognition inside their hospitals, SWs with this recognition were 6.08 times more likely to increase IPC practice compliance among SWs. As WHO reports, these groups are marginalized, stigmatized, and violated by members of society,^46^ which could be reducing the compliance or good level of IPC practice. In the real world, proper social recognition entails treating SWs with dignity and respect for their work, which in turn encourages them to follow IPC guidelines.^46^ Consequently, this study suggested that giving SWs positive social acknowledgment could boost IPC adherence. Because as compared to the other factors, it is the most often attributed factor for sanitary workers’ compliance with IPC practices.

In the meantime, SWs with inadequate PPE availability and supply were more likely to reduce the compliance of IPC practices by a factor of 20% when compared to those had adequate PPE availability and supply in their hospitals. On the other hand, it means that adequate PPE availability and supply were more likely to increase the compliance of IPC practices. This finding is consistent with the findings of the previous study, which found inadequate PPE contributed on poor IPC practice, where it has been two times more likely to have poor IPC measure than those who have good IPC practice.^41^ This is because PPE availability and supply are one of the primary applications for IPC implementations. Conversely, SWs without behavioral characteristics such a sleeping disorder saw a 58% decrease in IPC practice compliance compared to those who did. The study also found that SWs who did not use substances like chewing khat were more likely to increase IPC practice compliance compared to their peers. This result is in line with the earlier study's findings, which showed that workers who chewed khat habitually were more than twice as likely to violate IPC procedures than their peers.^41^

In a similar manner, the SWs working in Shift 2 (working afternoon) of the job rotation were more likely to have decrease compliance of IPC practice by 40% as compared to Shift 1, that is, those working in the morning during the study period. This might be the result of a busy morning with minimal focus on IPC protocols, but more standard‐based precautions being taken in the morning than in the afternoon, like medical waste management and other aspects of sanitation. This can lead to hospital workers disobeying IPC protocols during the study period. This study also found that SWs working as contact in the hospitals were more likely to decrease the compliance of IPC practice by 59% as compared to permanent SWs. The may be the result of inadequate training and orientation; contract workers are recruited for short periods of time to cover gaps left by departing SWs or mothers’ leaves. In addition, compared to those who received IPC instruction, those who did not receive OHS training were 70% more likely to have low IPC practice. This suggests that providing IPC training improves SWs’ adherence to IPC procedures. This finding is consistent with two studies, where workers who did not undergo training in IPC were almost three times more likely to have poor IPC measure than others.^41^ The other study also found that individual who had training on IPC practice were more likely to improve compliance with IPC practice nearly three times.^40^

The current study also assessed the core component of IPC practice among selected public hospitals with the accordance of WHO guideline.^29^ These hospitals subsequently implemented the overall core component IPC practice only by three‐fifths. This suggests that among these hospitals, two‐fifths (39.18%) of IPC services remain unsolved (Figure 1). The first of these components that have evaluated was the IPC's implementation. According to IPC experts’ feedback, the adherence to IPC practice among hospitals was inadequate (Supporting Information S1: Figure 1). In a broader sense, IPC implementation refers to designating an IPC coordinator, assigning separate funding and supplies, and provide sufficient and comfortable PPE. In the real world, having an autonomous budget for the IPC department and providing staff with appropriate, comfortable PPE can improve the department's overall performance, the safety environment, and patient care. However, the criticism found from the experts indicates that this is because the senior management of the structures has not demonstrated with clear commitment based on national targets.

The second core component was adaptation of IPC guideline at hospital level, which was inadequate (Figure 2). The observed gaps can be attributed to hospitals’ failure to implement IPC guidelines for both standard and transmission precautions. This is in line with a previous study conducted at a hospital in south Ethiopia, which found that less than half of hospitals had access to IPC guidelines.^40^ The third core component is providing IPC training practice for the staffs within the hospitals, which was less than half percent (Supporting Information S1: Figure 2). It became clear that about half of the institutions under evaluation did not provide IPC teaching or instruction to patients, visitors, or patients’ families. It is in line with data from the South Ethiopia Hospital, which showed that hospital ignorance of IPC guidelines was one of the most common problems in the hospitals.^40^

The fourth core component of IPC was conducting HAIs surveillance, where its compliance among the selected public hospitals was inadequate (Supporting Information S1: Figure 3). This nonadherence happened due to lack of unclear structure in place for surveillance and lack of surveillance priorities to identify which HAIs lack a defined monitoring plan for infections. The fifth core component was multimodal strategies for IPC intervention, where compliance of this practice was almost three‐fifths (Supporting Information S1: Figure 4). This core is crucial for intervening in IPC practice, but it is failed to practice because of failure of IPC system change, IPC instruction and training, IPC monitoring and feedback, communications, and reminders of IPC components.

The sixth core component was monitoring and providing feedback, where the compliance of this activities among public hospitals was inadequate (Supporting Information S1: Figure 5). Due to the absence of qualified staff in charge of overseeing and auditing IPC procedures, input about hand hygiene compliance and the overall performance of the IPC, as well as the procedures and metrics that the hospitals monitor, this core is inadequate. The seventh core component was preventive and initiative of employees, including SWs from infections and noninfections, which was very quiet (Supporting Information S1: Figure 6). The reasons for this were due to lack of national standards or a standard tool for staffing needs assessment including WHO workload and staffing indicators. This might have happened due to the overpopulation of patients in the hospital, due to which there is no proper amount of space between patient beds, which should be >1 m. The final or the eighth core component was building environmental condition for IPC activities, which was inadequate (Figure 4). Lack of drinking water, hand washing facilities, and sterilized, disinfected, and sterilizing containers were the gaps for these issues, which go counter to WHO recommendations.^29^

Regarding discussion of statistical analysis, at first time of the analysis, the VIF was performed to measure multicollinearity of independent variables. Accordingly, it was 4.18, indicating it is acceptable model b/s it is <10 according to HL^38^ explanation. The final random‐effect model (Model 3) of multilevel ordinal logistic regression analysis found the overall variance of compliance with IPC practice between SW from public hospitals to other public hospitals was 93.71% (Table 2). According to Rutala and Weber^43^ criteria, this shows that the evidence regarding IPC compliance among these hospitals has excellent reliability. Implying that the findings of the research indicate both individuals and hospitals must increase their share of these groups, to achieve advanced IPC practice in accordance with WHO guidelines.

## 5 STREGNTH AND LIMITATION OF THE STUDY

### 5.1 Strength of the study

The current study has several strengths: these obtained from both participants, namely from sanitary workers along with IPC experts. As a result, the concrete information was produced from Eastern Ethiopia's selected public hospitals that may serve as a future point of reference for similar research. The study also used an adequate number of samples and simple sampling procedures to lessen the possibility of sampling bias resulting from the selection of a sample of respondents from a diverse. Additionally, the study was able to estimate the components of IPC practice among workers as well as among public hospitals that assist the other scholars easily both adherence and nonadherence simultaneously, which could help hospitals for further improvement. Moreover, the research offers compelling evidence supporting the development of research hypotheses on IPC practice among sanitary workers and descriptive analysis.

### 5.2 Limitation of the study

This study has certain limitations. Among the limitations is since the current study primarily focuses on cross‐sectional studies, it cannot assess cause‐and‐effect relationship of IPC compliance. Thus, to maintain this, longitudinal study or an experimental study would be suggested. The second drawback is that, even while we have faith in the knowledge of IPC professionals, participant feedback—particularly that of IPC experts—may not be entirely indicative of the current status of IPC practice or its underlying issues. As a result, it might or might not lead to high or low reporting findings, which could cause information bias in the present investigation.

## 6 CONCLUSION

The overall study concluded that SWs working in selected public hospitals had inadequate IPC procedures. According to this study, IPC practices were related to training, understanding of using PPE, using addictive substances, job stress, workload every working hour, and providing social recognition for SWs. Consequently, research suggests that provide training, enhancing IPC knowledge, reducing work‐related stress and negative social recognition are the most determinant factors for improving IPC practice. The study also advised that maintaining standard procedures like PPE use and making them available to staff members should be encouraged. The study also was recommended to consider the type of work, appropriately rotate responsibilities, offer training, reduce stress, and work no more than 8 h every day to improve IPC practice.

In addition, the expert assessment revealed that the hospitals are adhering to the IPC core components practice with low status as compared to WHO indicators. As the assessment identified, there was no program was being executed, no one was being trained, no guidelines were in place and no one was performing HAIs. The study also found that there was no monitoring or feedback being provided, staffing was inadequate, and no IPC environment was being built were the main failure of IPC core practice. Therefore, the hospitals should have a responsible for improving the current IPC practice inline to national and WHO IPC guidle. The study also advised that good collaborative partnership or any project for future sustainable prevention and management of infections, which is very significant to meet safety, efficacy, patient contentedness, timeliness, efficiency, and equality of quality in hospitals.

## AUTHOR CONTRIBUTIONS

Sina T. Tolera, Tesfaye Gobena, Nega Aaaefa, and Abraham Geremew developed the idea, conceptualized, and theorized the evidence. Sina T. Tolera, Tesfaye Gobena, Nega Aaaefa, and Abraham Geremew gathered the data from the selected hospitals, analyzed data, validated, and visualized the data. Sina T. Tolera and Elka Toseva compiled data and edited the overall manuscript. All authors have read and approved the final version of the manuscript, had full access to all of the data in this study, and take complete responsibility for the integrity of the data and the accuracy of the data analysis.

## CONFLICT OF INTEREST STATEMENT

The authors declare no conflict of interest.

## TRANSPARENCY STATEMENT

The lead author Sina T. Tolera affirms that this manuscript is an honest, accurate, and transparent account of the study being reported; that no important aspects of the study have been omitted; and that any discrepancies from the study as planned (and, if relevant, registered) have been explained.

## Supporting information

Supporting Information

## Data Availability

The authors confirm that the data supporting the findings of this study are available as Supporting Information materials.
