# Supplementary material for: Compliance and determinants of infection prevention and control practices among sanitary workers in public hospitals, Eastern Ethiopia: A cross‐sectional study
Source: Health Sci Rep. 2024 Aug 23;7(8):e2318. doi: 10.1002/hsr2.2318 (PMC11342045; doi:10.1002/hsr2.2318)
Supplement: Supplementary file 1 — Supporting Information [file HSR2-7-e2318-s001.docx]

**Supplementary Materials**

Sup Figure 1 Non-compliance of IPC by program among selected public hospitals, 2023

Sup. Figure 2 Non-compliance of IPC by training and education of IPC among selected hospitals, 2023

Sup- Figure 3 Non-compliance of IPC by conducting Hospital Acquired Infections among public hospitals

Sup. Figure 4 Non-compliance of IPC by Initiating multimodal strategies among public hospitals, 2023

Sup. Figure 5- Non-compliance of IPC by conducting monitoring and feedback among selected hospitals

Sup. Figure 6 Non-compliance of IPC by preventing and initiation of staffing among selected hospitals, 2023
